# Transcatheter Aortic Valve Replacement Prognostication with Augmented Mean Arterial Pressure

**DOI:** 10.3390/jcdd10050192

**Published:** 2023-04-26

**Authors:** Chieh-Ju Chao, Pradyumna Agasthi, Amith R. Seri, Timothy Barry, Anusha Shanbhag, Yuxiang Wang, Mackram F. Eleid, David Fortuin, John P. Sweeney, Peter Pollak, Abdallah El Sabbagh, Steven J. Lester, William K. Freeman, Tasneem Z. Naqvi, David R. Holmes, Christopher P. Appleton, Reza Arsanjani

**Affiliations:** 1 Department of Cardiovascular Diseases, Mayo Clinic Arizona, Scottsdale, AZ 85259, USA; 2 Department of Cardiovascular Medicine, Mayo Clinic Rochester, Rochester, MN 55905, USA; 3 Department of Cardiovascular Diseases, Mayo Clinic Florida, Jacksonville, FL 32224, USA

**Keywords:** aortic valve stenosis, transcatheter aortic valve replacement (TAVR), STS risk score, augmented mean arterial pressure, mortality

## Abstract

Background: Post-transcatheter aortic valve replacement (TAVR) patient outcome is an important research topic. To accurately assess post-TAVR mortality, we examined a family of new echo parameters (augmented systolic blood pressure (AugSBP) and arterial mean pressure (AugMAP)) derived from blood pressure and aortic valve gradients. Methods: Patients in the Mayo Clinic National Cardiovascular Diseases Registry-TAVR database who underwent TAVR between 1 January 2012 and 30 June 2017 were identified to retrieve baseline clinical, echocardiographic and mortality data. AugSBP, AugMAP and valvulo-arterial impedance (Zva) (Zva) were evaluated using Cox regression. Receiver operating characteristic curve analysis and the c-index were used to assess the model performance against the Society of Thoracic Surgeons (STS) risk score. Results: The final cohort contained 974 patients with a mean age of 81.4 ± 8.3 years old, and 56.6% were male. The mean STS risk score was 8.2 ± 5.2. The median follow-up duration was 354 days, and the one-year all-cause mortality rate was 14.2%. Both univariate and multivariate Cox regression showed that AugSBP and AugMAP parameters were independent predictors for intermediate-term post-TAVR mortality (all *p* < 0.0001). AugMAP1 < 102.5 mmHg was associated with a 3-fold-increased risk of all-cause mortality 1-year post-TAVR (hazard ratio 3.0, 95%confidence interval 2.0–4.5, *p* < 0.0001). A univariate model of AugMAP1 surpassed the STS score model in predicting intermediate-term post-TAVR mortality (area under the curve: 0.700 vs. 0.587, *p* = 0.005; c-index: 0.681 vs. 0.585, *p* = 0.001). Conclusions: Augmented mean arterial pressure provides clinicians with a simple but effective approach to quickly identify patients at risk and potentially improve post-TAVR prognosis.

## 1. Background/Introduction

The success of transcatheter aortic valve replacement (TAVR) has substantially changed the landscape of managing aortic valve disease [1,2,3]. With the expansion of TAVR indications, it is anticipated that more TAVR procedures will be performed in the foreseeable future [4]. Given the underlying comorbidities of this patient population, post-TAVR outcome studies have gained significant attention [5,6,7,8,9,10,11,12]. The Society of Thoracic Surgeons (STS) risk score has been reported to be a strong predictor for short- and long-term post-TAVR prognosis, though it was originally designed to risk-stratify patients for surgical aortic valve replacement [13,14,15]. In addition, many studies have reported valvulo-arterial impedance (Zva) as a predictor for post-TAVR prognosis [8,9,10,16]. High Zva is associated with worse quality of life and exercise performance at one-year post-TAVR [10], while there are inconsistent results in predicting long-term mortality [17,18,19]. Nagura et al. suggested that Zva is sensitive to stroke volume index (SVi) change but not the arterial load. The potential measuring error from the stroke volume index can be magnified in Zva calculation, especially in patients with low-flow status [18]. To assess intermediate-term post-TAVR mortality, our group previously reported the cardiac power index and gradient-adjusted cardiac power index as good predictors of the endpoint [6,7]. Augmented blood pressure parameters were generated by calculating the gradient-adjusted cardiac power index by adding a transvalvular gradient (the mean transvalvular gradient or instantaneous peak transvalvular gradient) to the systolic blood pressure. This process is conceptually close to the summation of valvular and arterial load, which is the numerator of the Zva formula. Therefore, augmented blood pressure parameters can be viewed as a revised version of Zva by removing the component of SVi from it. In this context, we aimed to investigate the prognostic value of augmented blood pressure parameters in TAVR patients and compare them to Zva and the STS risk score. We hypothesized that augmented systolic blood pressure (AugSBP) and augmented mean arterial pressure (AugMAP) parameters are associated with intermediate-term all-cause mortality in patients who underwent a TAVR procedure.

## 2. Materials and Methods

### 2.1. Study Population, Baseline Demographics and Clinical Data

A chart review was conducted on patients in the Mayo Clinic National Cardiovascular Diseases Registry (NCDR)-TAVR database, which included patients from three major academic medical centers located in Rochester, MN, Phoenix, AZ, and Jacksonville, FL. We identified all patients aged ≥18 years who underwent TAVR between 1 January 2012 and 30 June 2017. Baseline demographics, lab data, device data, STS risk score and follow-up data were directly extracted from the database. Patients who had prior AVR (TAVR or SAVR) procedure(s) were excluded. Low-flow, low-gradient severe AS was defined as SVi < 35 mL/m^2^; AVA < 1 cm^2^ with AV peak velocity < 4 m/s or mean AV gradient < 40 mmHg. The Institutional Review Board at Mayo Clinic approved the study protocol, and all the patients provided research authorization to utilize their medical information.

### 2.2. Baseline Transthoracic Echocardiography

Baseline transthoracic echocardiography (TTE) with 2-dimension imaging and Doppler were performed pre-procedure using commercially available ultrasound scanners (Philips iE33; Phillips Medical Systems, Andover, MA, USA; GE Vivid E9, GE Healthcare, Milwaukee, WI, USA). All echocardiograms were measured and interpreted according to guidelines [20,21,22]. Offline measurements of the images were obtained using the ProSolv Cardiovascular Analyzer 3.0 (ProSolv Cardiovascular Inc., Indianapolis, IN, USA). The transaortic valvular flow rate (Q) was calculated according to the formula reported by Namasivayam et al. [23].

### 2.3. Calculation of Augmented Systolic Blood Pressure, Mean Arterial Pressure and Valvulo-Arterial Impedance

Noninvasive blood pressure measured at baseline TTE was used to calculate augmented blood pressure parameters. The SVi was calculated using the quantitative Doppler method. In patients with atrial fibrillation, the highest mean transvalvular gradient was used. The augmented blood pressure calculation formulas used are described as follows:

(1) Augmented SBP1 (AugSBP1): the mean aortic valve gradient (mean AVG) was added to systolic blood pressure (Equation (2)), and augmented MAP1 (AugMAP1) was calculated by replacing the SBP with augmented SBP1 in the MAP formula (Equation (3));
(2) Augmented SBP2 (AugSBP2): the aortic valve maximal instantaneous gradient was added to systolic blood pressure (Equation (4)), and augmented MAP2 (AugMAP2) was calculated by replacing the SBP with augmented SBP2 (Equation (5));
(3) Augmented MAP3 (AugMAP3): the aortic valve mean gradient was added to mean arterial pressure (Equation (6)) [6]. Zva was calculated according to the standard formula by dividing the sum of the systolic blood pressure and mean transvalvular gradient by stroke volume index (SVi) [18] (Equation (7)).

(1) $$\mathrm{MAP}=\left(\mathrm{SBP}+2\times \;\mathrm{DBP}\right)/3$$

(2) Augmented SBP1=mean AVG+SBP

(3) $$\mathrm{Augmented}\;\mathrm{MAP}1=\left(\mathrm{AugSBP}1+2\times \mathrm{DBP}\right)/3\;$$

(4) Augmented SBP2=maxinstantaneous AVG+SBP

(5) $$\mathrm{Augmented}\;\mathrm{MAP}2=\left(\mathrm{AugSBP}2+2\times \mathrm{DBP}\right)/3$$

(6) Augmented MAP3=MAP+mean AVG

(7) $$\mathrm{Zva}=\left(\mathrm{SBP}+\mathrm{mean}\;\mathrm{AVG}\right)/\mathrm{SVi}$$

### 2.4. Statistical Analysis

Patients were grouped into alive versus deceased groups, and higher AugMAP (≥median AugMAP1) versus lower AugMAP (<median AugMAP1) groups and analyzed accordingly. All the two-group comparisons were summarized as alive versus deceased groups if not otherwise specified. Continuous variables were summarized as mean ± standard deviation. The differences among groups were evaluated with the Student *t*-test and Mann–Whitney U test for normally and non-normally distributed data, respectively. Categorical variables were expressed as counts and percentages, and differences among groups were evaluated with the chi-square test. Kaplan–Meier survival curve and Cox regression were used for survival analysis; the median AugSBP1 and AugMAP1 were used as the cutoffs to stratify the patients for Kaplan–Meier analysis. The augmented blood pressure measurements, Zva and the STS risk score were used to develop univariate Cox regression models separately. In multivariate Cox regression analysis, two multivariate models were created. For model 1, variables were adjusted for STS risk score, right atrial (RA) pressure, right ventricular systolic pressure (RVSP) and albumin level; the variables already involved in the calculation of STS risk score (e.g., age, sex, hemodialysis, tricuspid regurgitation, etc.) were not used. Model 2 excluded STS risk score but included the variables in its calculation (Python lifelines 0.26.3). The receiver operating characteristics (ROC) curve with the area under the curve (AUC) and the c-index were used to assess the performance of each univariate model against the STS risk score model. We used the bootstrap method to estimate the 95% confidence interval (95%CI) of AUC and c-index for each univariate model. DeLong’s test was used to assess the differences in AUC, and a paired Student *t*-test was used to evaluate the differences in the c-indices [24]. A *p*-value of less than 0.05 was used as the cutoff of statistical significance for all the hypotheses. All the analyses were performed in Python version 3.7.10. 

### 2.5. Patient and Public Involvement

It was not appropriate or possible to involve patients or the public in the design, conducting, reporting, or dissemination plans of our research.

## 3. Results

### 3.1. Study Population and Baseline Demographics

A total of 1071 patients were initially identified. A total of 974 patients were included for the final analysis after excluding 97 patients who had prior aortic valve replacement (TAVR or SAVR). The mean age was 81.4 ± 8.3 years old. In total, 56.6% were male (*n* = 551), and 97.3% (*n* = 947) were white. The STS risk score was available for all patients (*n* = 974), with a mean of 8.2 ± 5.2. The median follow-up duration was 354 days (interquartile range 51–378 days), and the one-year all-cause mortality rate was 14.2% (*n* = 139). The median time from baseline TTE to TAVR was 1.5 months. Systolic blood pressure (alive versus deceased: 130.9 ± 21.4 mmHg vs. 119.1 ± 20.0 mmHg, *p* < 0.0001), diastolic blood pressure (68.9 ± 13.1 mmHg vs. 62.3 ± 12.3 mmHg, *p* < 0.0001) and mean arterial pressure (89.5 ± 13.4 mmHg vs. 81.2 ± 13.0 mmHg, *p* < 0.0001) were significantly higher in the alive group. Detailed data are summarized in Table 1. The additional group analysis (≥median AugMAP1 versus < median AugMAP1) is summarized in Supplemental Table S1.

### 3.2. Augmented Blood Pressure, Echocardiography and Zva Measurements

There was no significant difference in LV ejection fraction (57.8 ± 12.8% vs. 55.9 ± 14.5%, *p* = 0.221), mean aortic valve gradient (44.2 ± 13.1 mmHg vs. 42.7 ± 12.6 mmHg, *p* = 0.199), maximal aortic valve instantaneous gradient (72.1 ± 21.1 mmHg vs. 69.1 ± 21.0 mmHg, *p* = 0.080) and stroke volume index (43.7 ± 9.8 mL/m^2^ vs. 42.8 ± 9.7 mL/m^2^, *p* = 0.123). Both right atrial pressure (7.2 ± 3.8 mmHg vs. 8.9 ± 5.2 mmHg, *p* = 0.0004) and right ventricular systolic pressure (41.0 ± 13.2 mmHg vs. 45.4 ± 18.5 mmHg, *p* = 0.0097) were significantly higher in the deceased group.

**Figure 1:**
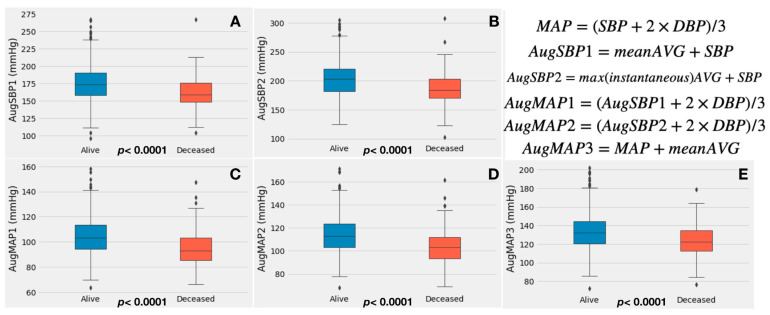
Box plot of augmented blood pressure measurements. Panels (**A**–**E**) demonstrate the box plot of each augmented blood pressure parameter. Augmented blood pressure parameters were significantly higher in alive patients compared to deceased patients (all *p* < 0.0001). The formulas used to calculate each parameter are listed in the upper-right corner. Rhombus symbol represents cases distributed beyond 2.5 standard deviations.

Regarding augmented blood pressure measurements, both AugSBP1 (174.9 ± 25.6 mmHg vs. 161.5 ± 23.9 mmHg, *p* < 0.0001) and AugSBP2 (203.0 ± 30.4 mmHg vs. 187.2 ± 30.0 mmHg, *p* < 0.0001) were significantly higher in the alive group. There were similar findings for AugMAP1 (104.1 ± 14.3 mmHg vs. 95.3 ± 13.7, *p* < 0.0001), AugMAP2 (113.3 ± 15.3 mmHg vs. 103.8 ± 15.1 mmHg, *p* < 0.0001) and AugMAP3 (133.5 ± 19.3 vs. 123.6 ± 18.1, *p* < 0.0001). Box plots were used to visualize the augmented blood pressure data (Figure 1). A higher Zva was also observed in the alive group (4.2 ± 1.1 mmHg ml^−1^ m^−2^ vs. 3.9 ± 0.9, *p* = 0.023).

### 3.3. Kaplan–Meier Analysis and Cox Regression

**Figure 2:**
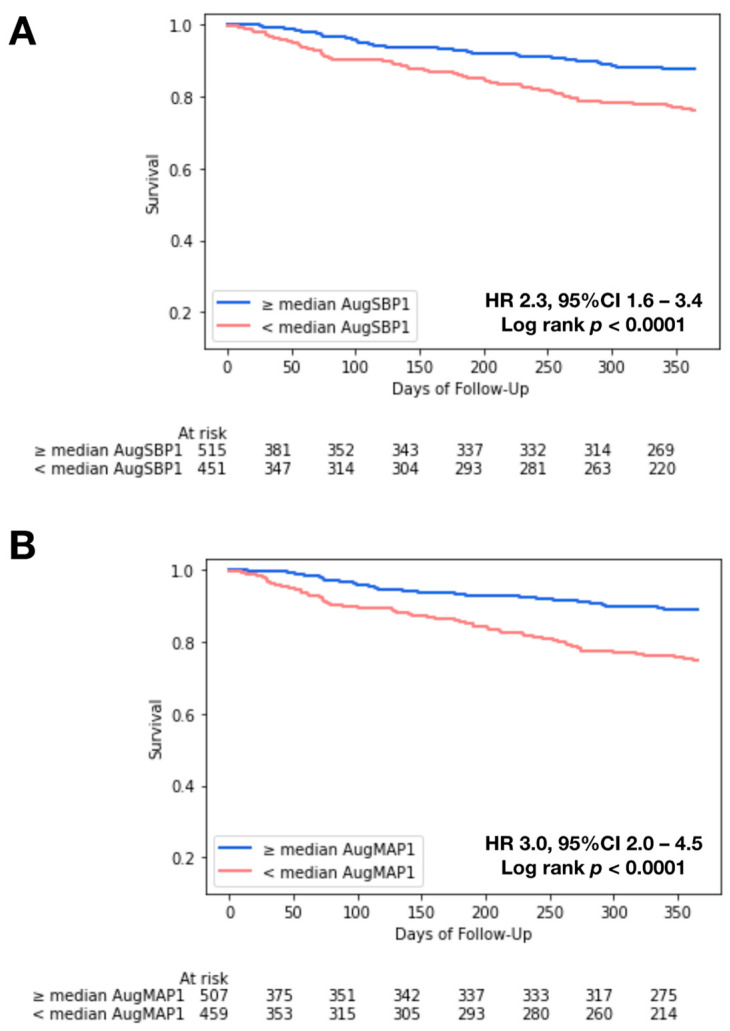
Kaplan–Meier survival curve analysis. Panel (**A**). Patients with ≥median AugSBP1 had significantly better survival compared to patients with <median AugSBP1; the median AugSBP1 was 171 mmHg (HR 2.3, 95%CI: 1.6–3.4, log-rank *p* < 0.0001). Panel (**B**). Patients with ≥median AugMAP1 had significantly better survival compared to patients with <median AugMAP1; the median AugMAP1 was 102.5 mmHg (HR 3.0, 95%CI: 2.0–4.5, log-rank *p* < 0.0001).

In Kaplan–Meier analysis, we observed significant survival differences when stratifying patients by the median AugSBP1 (171 mmHg; HR: 2.3, 95%CI 1.6–3.4, log-rank *p* < 0.0001) and the median AugMAP1 (102.5 mmHg; HR: 3.0, 95%CI: 2.0–4.5, log-rank *p*< 0.0001) (Figure 2A,B). Similar survival differences were observed in patients with low-flow, low-gradient AS (*n* = 85) (Supplemental Figure S1). In univariate Cox regression, AugSBP1, AugSBP2, AugMAP1, AugMAP2 and AugMAP3 were independently associated with intermediate-term all-cause mortality (all *p* < 0.0001). In addition, Zva, current dialysis, Afib/Aflutter, hemoglobin level, right atrial (RA) pressure, right ventricular systolic pressure (RVSP), ≥moderate TR and albumin level were also independent predictors for all-cause mortality in univariate regression, while stroke volume index was not associated with intermediate-term mortality (HR 0.99, 95%CI: 0.97–1.01, *p* = 0.3475). The associations of AugSBP and AugMAP parameters remained significant after adjusting for the STS risk score and other clinical risk factors in model 1 and model 2 (all *p* < 0.0001). Table 2 summarizes the Cox regression results.

### 3.4. Performance of Univariate Cox Regression Models

**Figure 3:**
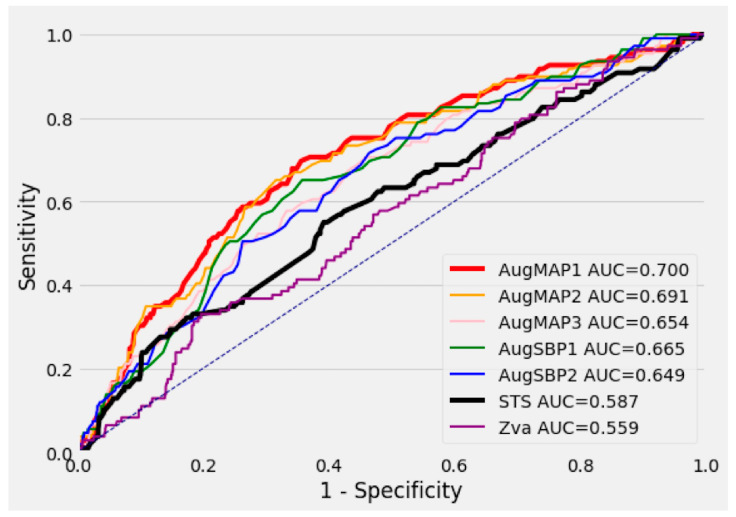
The ROC curves of all the single-parameter prediction models against the STS risk score model. The ROC curves of all the single-parameter prediction models against the STS risk score model (AUC 0.587, 95%CI 0.521–0.649). The AugMAP1 model had the best performance (AUC 0.700, 95%CI 0.646–0.750, *p* = 0.005), followed by the AugMAP2 model (AUC 0.691, 95%CI 0.636–0.743, *p* = 0.009). The rest of the augmented blood pressure (AugMAP3, AugSBP1, AugSBP2) parameters were comparable to the performance of the STS risk score (larger AUC, no statistical significance). Valvulo-arterial impedance (Zva) had a smaller AUC than the STS risk score, but this was not statistically significant (AUC 0.559, 95%CI 0.502–0.611, *p* = 0.519). Blue dotted line is the identity line.

The STS score univariate Cox regression model (reference model) had an AUC of 0.587 (95%CI 0.521–0.649) in predicting intermediate-term mortality. Among all other univariate Cox regression models, the AugMAP1 model (AUC 0.700, 95%CI 0.646–0.750, *p* = 0.005) and AugMAP2 model (AUC 0.691, 95%CI 0.636–0.743, *p* = 0.009) significantly outperformed the STS score model. AugSBP1 (AUC 0.665, 95%CI 0.612–0.719, *p* = 0.052), AugSBP2 (AUC 0.649, 95%CI 0.599–0.704, *p* = 0.118) and AugMAP3 (AUC 0.654, 95%CI 0.597–0.709, *p* = 0.086) were comparable/non-inferior in predicting mortality when compared to the STS score model. The AUC for Zva (0.559, 95%CI 0.502–0.611, *p* = 0.519) was numerically smaller than the STS score; however, the difference was not statistically significant. Figure 3 demonstrates the ROC curve of each univariate Cox regression model. We also observed similar model performances using the c-index, with AugMAP1 being the best univariate model (c-index 0.681, 95%CI: 0.644–0.705, *p* = 0.001). Details are summarized in Table 3.

## 4. Discussion

In this retrospective study, we demonstrated that baseline augmented mean arterial pressure (AugMAP1) surpassed the STS risk score in predicting intermediate-term post-TAVR all-cause mortality. To the best of our knowledge, this is the first application of augmented blood pressure parameters in assessing post-TAVR mortality. Our findings suggest that AugMAP1, as a surrogate marker of cardiac contractile function against systemic afterload in aortic stenosis patients, is closely associated with post-TAVR mortality and superior to the STS risk score. AugMAP is a simple but effective approach clinicians may employ to quickly identify high-risk patients and can potentially improve post-TAVR prognosis.

### 4.1. The Physiological Significance of Augmented Blood Pressure

When calculating the augmented blood pressure parameters, we assumed that adding either the mean or maximal instantaneous gradient to the systemic systolic blood pressure can reflect the true systolic pressure generated by the left ventricle [6]. Assuming a relatively small central venous pressure level, the MAP approximately equals the product of cardiac output and systemic vascular resistance (SVR). In this context, the MAP is proportional to cardiac output when the SVR is constant and can be considered the capability of the left ventricle to generate cardiac output against a given SVR. In the presence of aortic stenosis, an augmented MAP is the true mean pressure of the left ventricle in a cardiac cycle. We observed gradual decrease in model performance (AUC) of the univariate Cox models of AugMAP 1, 2 and 3, which reflected that any deviation (change in the formula) from AugMAP1 would lead to weaker associations between the parameter and post-TAVR mortality. Therefore, among the three AugMAP parameters, AugMAP1 should be considered the most accurate gauge of left ventricular contractile function against the systemic afterload and thus most closely associated with the post-TAVR prognosis. Additionally, for AugSBP1 and AugSBP2, while the AUC of each model was still larger than that of the STS model, the difference did not reach statistical significance as it did for AugMAP1 and AugMAP2. This can be explained by the same theory. Since the diastolic blood pressure component was removed from the formula, the AugSBP measurements were deviated to the end of systolic blood pressure, thus not reflecting the true MAP/contractile function.

### 4.2. An Overlooked Outcome Predictor: Augmented Blood Pressure

In both univariate and multivariate Cox regression analysis, all the augmented blood pressure parameters were independently associated with intermediate-term post-TAVR mortality. Notably, the AugMAP1 model had the best performance and surpassed the STS risk score in predicting intermediate-term mortality in the head-to-head ROC curve (*p* = 0.005) and c-index (*p* = 0.001) comparison. The STS score has been reported as a strong predictor for both short-and long-term post-TAVR mortality [13,14,15], so it was selected as the reference model in this study. Hemmann et al. reported that the STS score model had an AUC of 0.679 (95% CI: 0.610–0.748) in predicting intermediate-term post-TAVR mortality based on a cohort of 426 patients, and a performance superior to that of the EuroSCORE and EuroSCORE2 [14]. In another study with 3491 TAVR patients, the STS score model had an AUC of 0.61 (95% CI: 0.56–0.67) in predicting 30-day post-TAVR mortality [15]. Overall, the AUC of the STS score models in prior studies was in the range of 0.6–0.7, similar to the finding in our study (AUC 0.587, 95%CI 0.521–0.649). Furthermore, the performance of the univariate AugMAP1 model was not inferior to any of the STS score models above [13,14,15] and almost reached the same level as our machine learning model based on the same database [5]. Patients with an AugSBP1 or AugMAP1 below the median cutoff were also associated with a 2–3-fold-increased risk of mortality in Kaplan–Meier analysis (Figure 2A,B). The two parameters are simple, easy to calculate and accurate in stratifying post-TAVR prognosis, and only require a few variables as their input.

A possible concern in using this approach is that the parameter primarily relies on blood pressure measurements, which can vary from time to time, and suboptimal blood pressure control may cause higher blood pressure readings. In our cohort, all of SBP, DBP and MAP measurements were significantly higher in the alive group (all *p* < 0.0001), with the mean SBP in the alive group being reasonably controlled (130.9 ± 21.4 mmHg). There was no significant difference between the alive and deceased groups regarding antihypertensive medications. Earlier studies have shown that higher blood pressure/hypertension after the TAVR procedure is associated with better outcomes [25], in contrast to lower blood pressure [9]. Furthermore, patients who developed hypertension post-TAVR were found to have significantly improved cardiac output and stroke volume [25]. These patients likely had better cardiac contractile function reserved to generate higher blood pressure after relieving the valvular stenosis, in contrast to those with lower blood pressure [9]. While it is beyond the scope of this work to suggest an optimal blood pressure target for TAVR patients, the mean SBP and DBP of our alive patients were close to the lessened systolic blood pressure target (130–139 mmHg) for aortic stenosis patients suggested in a new European consensus statement [26].

### 4.3. Valvulo-Arterial Impedance (Zva) vs. AugMAP/AugSBP

In 2009, Hachicha et al. first demonstrated Zva was independently associated with mortality in aortic stenosis patients who underwent either surgical aortic valve replacement or medical therapy [27]. After that, Zva was evaluated in multiple studies as a post-TAVR outcome predictor [10,17,18,19,28,29]. A cohort study with 202 TAVR patients reported that baseline Zva was independently associated with 6-month all-cause mortality, but this conclusion was only based on univariate logistic regression and was not adjusted for other covariates [19]. In terms of longer-term mortality, Katsanos et al. showed that higher baseline Zva was independently associated with post-TAVR mortality at 2 years [17]. However, the study was based on a small patient cohort (*n* = 116) with a mortality endpoint in 21 (18%) patients. Another study based on the Optimized CathEter vAlvular iNtervention (OCEAN)-TAVI registry included 1004 patients but reported that post-TAVR Zva was not associated with two-year all-cause mortality in multivariate analysis; the correlation between baseline Zva and mortality was not assessed in this study [18]. Importantly, none of the above studies had used Zva to generate a univariate model and compared the performance with the STS risk score [17,18,19]. In the current study, baseline Zva was an independent predictor of intermediate-term post-TAVR mortality in univariate Cox regression, but not after adjusting for age, sex and the STS score. Furthermore, the univariate Zva model had the worst performance, while not statistically significant compared to the STS model (0.559 vs. 0.587, *p* = 0.519). Compared to the performance of augmented blood pressure measurements, the component of total load (SBP + mean AVG, equals to AugSBP1) is likely the key of Zva’s association with mortality in prior works [17,18,19]. Introducing stroke volume index as the denominator substantially canceled out the contribution of cardiac output from the total load term and can bring additional measuring error to this parameter, especially in patients with low-flow status [18]. Our true MAP prognosis theory anticipates that the best accuracy Zva could achieve was at the level of AugSBP1 (the numerator of the Zva formula). However, with the contribution and potential measurement error from the SVi component, Zva deviates more from AugMAP1, thus having a worse performance than AugSBP1. Our results comparing AugMAP and Zva also support that cardiac contractile function against the afterload is more important than the vascular load in determining post-TAVR prognosis.

### 4.4. Incorporating Augmented MAP in the Assessment of TAVR Patients

Compared to the STS risk score, which requires an input of more than 70 variables and relies on an online calculator after completing extensive workup [30], our models with AugMAP and AugSBP provide a simple but effective method that only requires two to three readily available variables (SBP, DBP and mean AVG) to make a superior prediction. The median cutoffs (AugSBP1: 171 mmHg and AugMAP1: 102.5 mmHg) can be easily calculated at the bedside to provide real-time assessment, which may facilitate workup and decision making, and can potentially improve post-TAVR prognosis by identifying high-risk patients.

## 5. Conclusions

As a single parameter, augmented mean arterial pressure (AugMAP1) surpassed the STS risk score in predicting intermediate-term post-TAVR mortality. Augmented blood pressure parameters provide a simple but effective approach clinicians may employ to quickly estimate the clinical outcome of TAVR patients and can potentially improve post-TAVR prognosis by early identifying high-risk patients.

## 6. Limitations

This study is limited by its retrospective nature, and external validation is not available for the findings. To maintain a sufficient sample size, we did not further divide our patient cohort into subgroups for ROC analysis. To allow comparison with prior studies, we mainly used ROC-AUC as the metric of model performance, but the c-index results also demonstrated similar findings. Concerning the period of TAVR procedures, most of the patients in this cohort were considered to be at intermediate-to-high risk, so low-risk patients may not be well represented in this development cohort. Only a small portion of patients had ≥ moderate AR, which limits the applicability of AugMAP in patients with clinically significant AR. The time from the baseline TTE/blood pressure measurement to the TAVR procedure varied among patients, and the potential changes occurring between these two points were not considered. However, the median time from baseline TTE to TAVR was relatively short (1.5 months) for significant hemodynamic changes. While our approach eliminated the contribution of stroke volume index, the component of systolic blood pressure and transvalvular gradient in this parameter still inherited the intrinsic limitation of Zva [31]. Additionally, invasive hemodynamic measurements were not available to validate the correlations between non-invasively measured augmented MAP and the invasive MAP, which is a potential direction for future studies. In addition, only all-cause mortality was available as an endpoint; whether augmented blood pressure parameters were related to other endpoints (cardiovascular mortality, heart failure hospitalization, etc.) shall be deferred to future studies.

## Figures and Tables

**Table 1:** Baseline Characteristics of Patients.

Variables	Alive	Deceased	Total	*p*-Value
	*N* = 835	*N* = 139	*N* = 974	
**Baseline Demographics**				
Age (year)	81.5 ± 8.1	80.7 ± 9.4	81.4 ± 8.3	0.302
Male sex (%)	469 (56.2%)	82 (59.0%)	551 (56.6%)	0.824
Caucasian race	814 (97.5%)	133 (95.7%)	947 (97.3%)	0.792
STS risk score	7.9 ± 5.1	9.8 ± 5.3	8.2 ± 5.2	<0.0001
Hypertension	702 (84.1%)	119 (85.6%)	821 (84.3%)	0.899
Diabetes mellitus	300 (35.9%)	54 (38.8%)	354 (36.3%)	0.803
Prior MI	200 (24.0%)	37 (26.6%)	237 (24.3%)	0.794
Prior CABG	208 (24.9%)	42 (30.2%)	250 (25.7%)	0.415
Prior stroke	75 (9.0%)	20 (14.4%)	95 (9.8%)	0.138
Prior PAD	421 (50.4%)	75 (54.0%)	496 (50.9%)	0.742
Current dialysis	26 (3.1%)	13 (9.4%)	39 (4.0%)	0.002
Atrial fibrillation/atrial flutter	322 (38.6%)	84 (60.4%)	406 (41.7%)	<0.0001
Permanent pacemaker	119 (14.3%)	25 (18.0%)	144 (14.8%)	0.517
Previous implantable cardioverter device	32 (3.8%)	6 (4.3%)	38 (3.9%)	0.963
NYHA class within 2 weeks				0.656
I	23 (2.8%)	7 (5.0%)	30 (3.1%)	
II	184 (22.0%)	27 (19.4%)	211 (21.7%)	
III	513 (61.4%)	80 (57.6%)	593 (60.9%)	
IV	115 (13.8%)	25 (18.0%)	140 (14.4%)	
Device type				0.315
Balloon-expandable valve	677 (81.1%)	105 (75.5%)	782 (80.3%)	
Self-expanding valve	158 (18.9%)	34 (24.5%)	192 (19.7%)	
Aortic valve morphology				0.029
Tricuspid	818 (98.0%)	138 (99.3%)	956 (99.0%)	
Bicuspid	8 (1.0%)	0 (0.0%)	0 (0.8%)	
**Augmented Blood Pressure Parameters**				
Heart rate (bpm)	70.3 ± 13.1	69.1 ± 12.7	70.2 ± 13.0	0.218
Systolic blood pressure (mmHg)	130.9 ± 21.4	119.1 ± 20.0	129.3 ± 21.6	<0.0001
Diastolic blood pressure (mmHg)	68.9 ± 13.1	62.3 ± 12.3	68.0 ± 13.2	<0.0001
Mean arterial blood pressure (mmHg)	89.5 ± 13.4	81.2 ± 13.0	88.3 ± 13.6	<0.0001
Aortic valve systolic mean gradient (mmHg)	44.2 ± 13.1	42.7 ± 12.6	44.0 ± 13.1	0.199
Aortic valve systolic maximal instantaneous gradient (mmHg)	72.1 ± 21.1	69.1 ± 21.0	71.7 ± 21.1	0.080
Aortic valve systolic peak velocity (m/s)	4.2 ± 0.6	4.1 ± 0.6	4.2 ± 0.6	0.139
Aortic valve systolic area (cm^2^)	0.8 ± 0.3	0.8 ± 0.3	0.8 ± 0.3	0.410
AugSBP1 (mmHg)	174.9 ± 25.6	161.5 ± 23.9	173.0 ± 25.8	<0.0001
AugSBP2 (mmHg)	203.0 ± 30.4	187.2 ± 30.0	200.7 ± 30.8	<0.0001
AugMAP1 (mmHg)	104.1 ± 14.3	95.3 ± 13.7	102.9 ± 14.6	<0.0001
AugMAP2 (mmHg)	113.3 ± 15.3	103.8 ± 15.1	111.9 ± 15.6	<0.0001
AugMAP3 (mmHg)	133.5 ± 19.3	123.6 ± 18.1	132.1 ± 19.5	<0.0001
Zva (mmHg mL^−1^ m^−2^)	4.2 ± 1.1	3.9 ± 0.9	4.2 ± 1.1	0.023
**Medications**				
Aspirin	122 (14.6%)	21 (15.1%)	143 (14.7%)	0.988
Beta blocker	583 (69.9%)	82 (63.1%)	665 (69.0%)	0.619
ACE inhibitor	147 (17.6%)	24 (18.5%)	171 (17.8%)	0.932
ARB	76 (9.1%)	11 (8.5%)	87 (9.0%)	0.403
**Labs**				
Hemoglobin (g/dL)	12.2 ± 1.8	11.4 ± 1.9	12.1 ± 1.8	<0.0001
Creatinine (mg/dL)	1.3 ± 1.0	1.7 ± 1.6	1.4 ± 1.1	<0.0001
Total albumin (g/dL)	4.1 ± 0.3	4.0 ± 0.4	4.1 ± 0.4	0.001
**Echocardiography Parameters**				
Left ventricular mass index (g/m^2^)	118.2 ± 32.0	121.1 ± 32.4	118.6 ± 32.1	0.173
Left ventricular ejection fraction(%)	57.8 ± 12.8	55.9 ± 14.5	57.5 ± 13.1	0.221
Estimated right atrial pressure (mmHg)	7.2 ± 3.8	8.9 ± 5.2	7.4 ± 4.1	0.0004
Right ventricular systolic pressure (mm Hg)	41.0 ± 13.2	45.4 ± 18.5	41.7 ± 14.2	0.010
Left ventricular internal systolic dimension (mm)	32.4 ± 8.3	34.2 ± 10.3	32.7 ± 8.7	0.092
Left ventricular internal end diastolic dimension (mm)	48.8 ± 7.0	49.6 ± 8.8	48.9 ± 7.3	0.203
Left ventricular stroke volume index (mL/m^2^)	43.7 ± 9.8	42.8 ± 9.7	43.6 ± 9.8	0.123
Transvalvular flow rate (Q, mL/s)	259.4 ± 71.2	261.9 ± 86.3	259.7 ± 73.4	0.309
Left ventricular cardiac output (L/min)	5.7 ± 1.3	5.3 ± 1.2	5.6 ± 1.3	0.003
Aortic valve systolic TVI (cm)	102.9 ± 19.8	98.8 ± 21.3	102.3 ± 20.0	0.024
≥Moderate aortic regurgitation	113 (14.9%)	14 (10.7%)	127 (14.3%)	0.530
≥Moderate mitral valve regurgitation	203 (24.3%)	38 (27.7%)	241(24.8%)	0.864
≥Moderate tricuspid valve regurgitation	193 (23.1%)	50 (36.0%)	243 (24.9%)	0.005

STS: Society of Thoracic Surgeons, MI: myocardial infarction, CABG: coronary artery bypass graft, PAD: peripheral artery disease, NYHA: New York Heart Association, SBP: systolic blood pressure, MAP: mean arterial pressure, Zva: valvulo-arterial impedance, ACE: angiotensin-converting enzyme, ARB: angiotensin receptor blocker, LV: left ventricle.

**Table 2:** Univariate Cox regression.

Covariate	HR (Per Unit Increase)	Lower 95%CI	Upper 95%CI	*p*-Value
Age	0.990	0.972	1.009	0.317
Sex	0.871	0.616	1.229	0.431
HTN	1.130	0.695	1.837	0.621
DM	1.085	0.766	1.537	0.647
Stroke	1.490	0.906	2.448	0.116
PriorMI	1.179	0.804	1.728	0.399
PAD	1.062	0.755	1.492	0.731
Current hemodialysis	2.383	1.316	4.313	0.004
Afib/Aflutter	2.213	1.564	3.131	<0.0001
Hgb (per unit increase)	0.800	0.725	0.882	<0.0001
RAP (per unit increase)	1.076	1.033	1.121	0.0004
RVSP (per unit increase)	1.020	1.009	1.031	0.0003
Creatinine (per unit increase)	1.172	1.066	1.289	0.001
Albumin (per unit increase)	0.420	0.280	0.632	<0.0001
≥Moderate MR	1.195	0.815	1.752	0.361
≥Moderate TR	1.885	1.321	2.690	0.0005
LVDD	1.097	0.921	1.306	0.300
STS risk score (per unit increase)	1.027	1.009	1.046	0.003
Q (per unit increase)	1.001	0.998	1.003	0.535
LVEF (per unit increase)	0.994	0.982	1.006	0.320
SBP (per unit increase)	0.971	0.962	0.981	<0.0001
DBP (per unit increase)	0.960	0.946	0.975	<0.0001
mean AVG (per unit increase)	0.990	0.977	1.003	0.145
AugMAP1 (per unit increase)	0.956	0.943	0.969	<0.0001
AugMAP2 (per unit increase)	0.960	0.948	0.972	<0.0001
AugMAP3 (per unit increase)	0.973	0.964	0.983	<0.0001
AugSBP1 (per unit increase)	0.978	0.971	0.986	<0.0001
AugSBP2 (per unit increase)	0.983	0.977	0.989	<0.0001
Zva (per unit increase)	0.819	0.674	0.996	0.046
Stroke volume index (per unit increase)	0.991	0.972	1.010	0.347
**Multivariate Cox Regression**				
**Covariate**	**HR (Per Unit Increase)**	**Lower 95%CI**	**Upper 95%CI**	***p*-Value**
Model 1 *				
AugMAP1 (per unit increase)	0.958	0.944	0.973	<0.0001
AugMAP2 (per unit increase)	0.960	0.947	0.974	<0.0001
AugMAP3 (per unit increase)	0.975	0.965	0.985	<0.0001
AugSBP1 (per unit increase)	0.979	0.970	0.987	<0.0001
AugSBP2 (per unit increase)	0.982	0.975	0.989	<0.0001
Zva (per unit increase)	0.760	0.618	0.934	0.009
Model 2 **				
AugMAP1 (per unit increase)	0.961	0.945	0.976	<0.0001
AugMAP2 (per unit increase)	0.961	0.948	0.976	<0.0001
AugMAP3 (per unit increase)	0.976	0.965	0.987	<0.0001
AugSBP1 (per unit increase)	0.979	0.971	0.988	<0.0001
AugSBP2 (per unit increase)	0.982	0.975	0.989	<0.0001
Zva (per unit increase)	0.756	0.610	0.936	0.010

HTN: hypertension, DM: diabetes mellitus, MI: myocardial infarction, PAD: peripheral arterial disease, Afib: atrial fibrillation, Aflutter: atrial flutter, MR: mitral regurgitation, TR: tricuspid regurgitation, Hgb: hemoglobin, RAP: right atrial pressure, RVSP: right ventricular systolic pressure, LVDD: left ventricular diastolic dysfunction, STS risk score: Society of Thoracic Surgeons risk score, LVEF: left ventricular ejection fraction, AugMAP: augmented mean arterial blood pressure, AugSBP: augmented systolic blood pressure, Zva: valvulo-arterial impedance. * Adjusted for STS risk score, estimated RA pressure, RVSP and albumin level (variables already included in the calculation of the STS risk score were not used as a covariate). ** Adjusted for age, sex, HTN, DM, stroke, MI, PAD, Afib/flutter, ≥moderate TR, ≥moderate MR, Hgb, creatinine, albumin, RAP and RVSP.

**Table 3:** C-indices of Univariate Models.

Univariate Model	C-Index	Lower 95%CI	Upper 95%CI	*p*-Value *
STS risk score	0.585	0.554	0.653	--
AugMAP1	0.681	0.644	0.705	0.001
AugMAP2	0.679	0.625	0.708	0.003
AugMAP3	0.647	0.545	0.704	0.031
AugSBP1	0.651	0.594	0.686	0.032
AugSBP2	0.645	0.584	0.662	0.050
Zva	0.552	0.499	0.653	0.033

STS: Society of Thoracic Surgeons, LVEF: left ventricular ejection fraction, AugMAP: augmented blood pressure, AugSBP: augmented systolic blood pressure, Zva: valvulo-arterial impedance. * Against STS risk score.

## Data Availability

Data are contained within the article or Supplementary Material.

## Supplementary Materials

The following supporting information can be downloaded at: https://www.mdpi.com/article/10.3390/jcdd10050192/s1, Figure S1: AugSBP1 and AugMAP1 in patients with low-flow, low-gradient severe aortic stenosis. Panel A. Patients with ≥ median AugSBP1 had better survival compared to patients with < median AugSBP1; the median of AugSBP1 is 171 mmHg (log-rank p= 0.066). Panel B. Patients with ≥ median AugMAP1 had significantly better survival compared to patients with < median AugMAP1; the median of AugMAP1 is 102.5 mmHg (log-rank p= 0.0008).; Table S1: Baseline Characteristics of Patients (Grouped by high vs. low AugMAP1).

## Abbreviations

AVG	aortic valve gradient
AugSBP	augmented systolic blood pressure
AugMAP	augmented mean arterial pressure
CO	cardiac output
DBP	diastolic blood pressure
MAP	mean arterial pressure
SBP	systolic blood pressure
Svi	stroke volume index
STS	Society of Thoracic Surgeons
SAVR	surgical aortic valve replacement
TTE	transthoracic echocardiography
TAVR	transcatheter aortic valve replacement
Zva	valvulo-arterial impedance
