# Supplementary material for: Transcatheter Aortic Valve Replacement Prognostication with Augmented Mean Arterial Pressure
Source: J Cardiovasc Dev Dis. 2023 Apr 26;10(5):192. doi: 10.3390/jcdd10050192 (PMC10218897; doi:10.3390/jcdd10050192)
Supplement: Supplementary file 1 [file jcdd-10-00192-s001.zip › jcdd-2341826-supplementary.pdf]

Supplementary materials

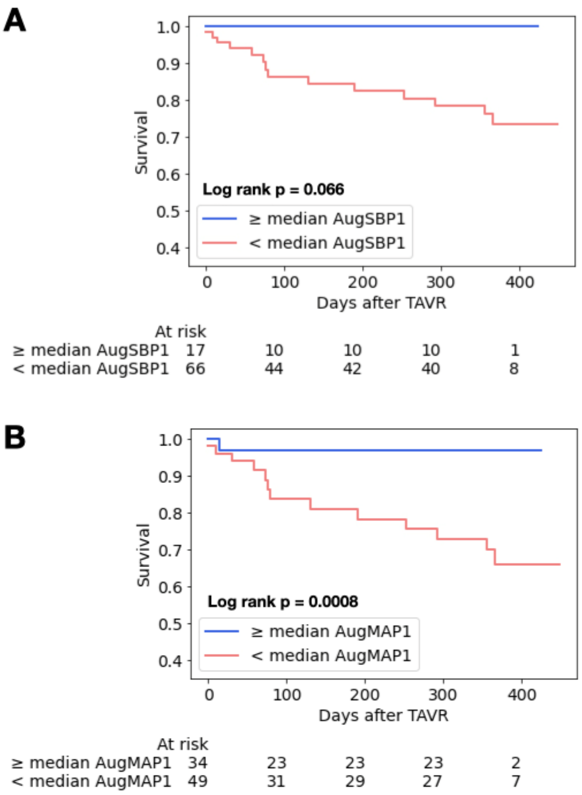

Figure S1. AugSBP1 and AugMAP1 in patients with low-flow, low-gradient severe aortic stenosis. Panel A. Patients with ≥ median AugSBP1 had better survival compared to patients with < median AugSBP1; the median of AugSBP1 is 171 mmHg (log-rank p= 0.066). Panel B. Patients with ≥ median AugMAP1 had significantly better survival compared to patients with < median AugMAP1; the median of AugMAP1 is 102.5 mmHg (log-rank p= 0.0008).

Table S1. Baseline Patient Characteristics (Grouped by high vs. low AugMAP1).

Group	Higher AugMAP1	Lower AugMAP1	Total	P value
**Variables**	**N=510**	**N=464**	**N=974**	
**Baseline Demographics**				
Age(yr)	81.4±8.0	81.4±8.6	81.4±8.3	0.84
Male sex (%)	259 (50.8%)	292 (62.9%)	551 (56.6%)	0.001
Caucasian race	497 (97.5%)	450 (97.0%)	947 (97.6%)	0.79
STS risk score	7.7±4.4	8.7±5.9	8.2±5.2	0.003
Hypertension	430 (84.3%)	391 (84.3%)	821 (84.3%)	1.00
Diabetes mellitus	172 (33.7%)	182 (39.2%)	354 (36.3%)	0.20
PriorMI	101 (19.8%)	136 (29.3%)	237 (24.3%)	0.003
Prior CABG	112 (22.0%)	138 (29.7%)	250 (25.7%)	0.021
Prior Stroke	39 (7.6%)	56 (12.1%)	95 (9.8%)	0.067
Prior PAD	238 (46.7%)	258 (55.6%)	496 (50.9%)	0.021
Current dialysis	23 (4.5%)	16 (3.4%)	39 (4.0%)	0.70
Atrial fib/flutter	185 (36.3%)	221 (47.6%)	406 (41.7%)	0.002
Permanent pacemaker	69 (13.5%)	75 (16.2%)	144 (14.8%)	0.51
Previous implantable cardioverter device	20 (3.9%)	18 (3.9%)	38 (3.9%)	1.00
NYHA class within 2 weeks				0.56

I	18 (3.5%)	12 (2.6%)	30 (3.1%)	
II	115 (22.5%)	96 (20.7%)	211 (21.7%)	
III	315 (61.8%)	278 (59.9%)	593 (60.9%)	
IV	62 (12.2%)	78 (16.8%)	140 (14.4%)	
Device Type				0.67
Balloon expandable vlave	415 (81.4%)	367 (79.1%)	782 (80.3%)	
Self expanding valve	95 (18.6%)	97 (20.9%)	192 (19.7%)	
AVDMorphology				
Tricuspid	500 (98.0%)	456 (98.3%)	956 (98.2%)	0.99
Bicuspid	4 (0.8%)	4 (0.9%)	8 (0.8%)	
Uncertain	6 (1.2%)	4 (0.9%)	10 (1.0%)	
**Augmented blood pressure parameters**				
Heart Rate	70.8±12.9	69.5±13.2	70.2±13.0	0.11
Systolic Blood Pressure	142.8±19.4	115.5±13.4	129.3±21.6	<0.0001
Diastolic Blood Pressure	76.9±11.0	58.9±8.2	68.0±13.2	<0.0001
Mean arterial blood pressure	98.8±9.9	77.8±7.4	88.3±13.6	<0.0001
Aortic vlave systolic mean gradient (mm Hg)	47.2±13.4	40.5±11.7	44.0±13.1	<0.0001
Aortic valve systolic maximal instantaneous gradient	76.7±21.4	66.3±19.4	71.7±21.1	<0.0001
Aortic valve systolic peak velocity by CWD	4.4±0.6	4.1±0.6	4.2±0.6	<0.0001
Aortic Valve Systolic Area by Velocity	0.8±0.2	0.9±0.3	0.9±0.3	0.0001
AugSBP1	189.9±22.2	156.0±16.2	173.0±25.8	<0.0001
AugSBP2	219.7±26.6	182.0±22.0	200.7±30.8	<0.0001
AugMAP1	114.5±9.8	91.3±7.6	102.9±14.6	<0.0001
AugMAP2	124.2±10.6	99.9±8.9	111.9±15.6	<0.0001
AugMAP3	146.0±14.8	118.3±12.5	132.1±19.5	<0.0001
Zva	4.5±1.0	3.8±1.0	4.2±1.1	<0.0001
**Medications**				
Aspirin	69 (13.5%)	74 (15.9%)	143 (14.7%)	0.56
Beta Blocker	351 (69.1%)	314 (68.9%)	665 (69.0%)	1.00
ACE Inhibitor	83 (16.3%)	88 (19.3%)	171 (17.8%)	0.65
ARB	59 (11.6%)	28 (6.1%)	87 (9.0%)	0.009
**Labs**				
Hemoglobin (g/dL)	12.3±1.8	11.8±1.8	12.1±1.8	<0.0001
Creatinine (mg/dL)	1.4±1.2	1.4±1.0	1.4±1.1	0.0004
Total albumin (g/dL)	4.1±0.4	4.1±0.4	4.1±0.4	0.22
**Echocardiography parameters**				
Left ventricular mass index (g/m^2)	227.8±65.8	226.6±65.4	227.2±65.6	0.90
Left ventricular ejection fraction(%)	59.1±12.1	55.8±13.9	57.5±13.1	0.0005
Estimated right atrial pressure (mmHg)	6.9±3.7	7.9±4.4	7.4±4.1	0.0005
Right ventricular systolic pressure (mm Hg)	41.0±13.7	42.3±14.7	41.7±14.2	0.15
Left ventricular internal systolic dimension	31.7±8.1	33.8±9.1	32.7±8.7	<0.0001
Left ventricular internal end diastolic dimension (mm)	48.0±6.9	49.8±7.6	48.9±7.3	<0.0001
Left ventricular stroke volume index (mL/m^2)	43.9±9.3	43.2±10.3	43.6±9.8	0.22
Transvalvular flow rate (Q, mL/s)	249.1±54.5	271.1±87.9	259.8±73.4	<0.0001
Left ventricular cardiac output (L/min)	5.7±1.3	5.5±1.3	5.6±1.3	0.03
Left ventricular internal end diastolic dimension	31.7±8.1	33.8±9.1	32.7±8.7	<0.0001
Aortic valve systolic TVI (cm)	106.7±19.7	97.7±19.4	102.3±20.0	<0.0001
≥ moderate aortic regurgitation	62 (13.0%)	65 (15.4%)	127 (14.3%)	0.0002
≥ moderate mitral valve regurgitation	107 (21.1%)	134 (29.0%)	241 (24.8%)	0.21
≥ moderate tricuspid valve regurgitation	116 (22.7%)	127 (27.4%)	243 (25.0%)	0.39

STS: Society of Thoracic Surgeons, MI: myocardial infarction, CABG: coronary artery bypass graft, PAD: peripheral artery disease, NYHA: New York Heart Association, SBP: systolic blood pressure, MAP: mean arterial pressure, Zva: Valvulo-arterial impedance, ACE: angiotensin converting enzyme, ARB: angiotensin recepter blocker, LV: left ventricle.
